# The RNA-binding protein hnRNPA2 regulates β-catenin protein expression and is overexpressed in prostate cancer

**DOI:** 10.4161/rna.28800

**Published:** 2014-04-24

**Authors:** Jacqueline Stockley, M Eugenia M Villasevil, Colin Nixon, Imran Ahmad, Hing Y Leung, Prabhakar Rajan

**Affiliations:** 1 Institute of Cancer Sciences; College of Medical, Veterinary, and Life Sciences; University of Glasgow; Cancer Research UK Beatson Institute; Bearsden, UK; 2 Cancer Research UK Beatson Institute; The Beatson Institute for Cancer Research; Bearsden, UK

**Keywords:** 3-UTR mRNA, *CTNNB1*, *HNRNPA2B1*, prostate cancer, β-catenin

## Abstract

Introduction

The RNA-binding protein hnRNPA2 (*HNRNPA2B1*) is upregulated in cancer, where it controls alternative pre-mRNA splicing of cancer-relevant genes. Cytoplasmic hnRNPA2 is reported in aggressive cancers, but is functionally uncharacterized. We explored the role of hnRNPA2 in prostate cancer (PCa). **Methods:** hnRNPA2 function/localization/expression in PCa was determined using biochemical approaches (colony forming/proliferation/luciferase reporter assays/flow cytometry/immunohistocytochemistry). Binding of hnRNPA2 within cancer-relevant 3′-UTR mRNAs was identified by bioinformatics. **Results:** RNAi-mediated knockdown of hnRNPA2 reduced colony forming and proliferation, while hnRNPA2 overexpression increased proliferation of PCa cells. Nuclear hnRNPA2 is overexpressed in high-grade clinical PCa, and is also observed in the cytoplasm in some cases. Ectopic expression of a predominantly cytoplasmic variant hnRNPA2-ΔRGG also increased PCa cell proliferation, suggesting that cytoplasmic hnRNPA2 may also be functionally relevant in PCa. Consistent with its known cytoplasmic roles, hnRNPA2 was associated with 3′-UTR mRNAs of several cancer-relevant mRNAs including β-catenin (*CTNNB1*). Both wild-type hnRNPA2 and hnRNPA2-ΔRGG act on *CTNNB1* 3′-UTR mRNA, increasing endogenous *CTNNB1* mRNA expression and β-catenin protein expression and nuclear localization. **Conclusion:** Nuclear and cytoplasmic hnRNPA2 are present in PCa and appear to be functionally important. Cytoplasmic hnRNPA2 may affect the cancer cell phenotype through 3′-UTR mRNA-mediated regulation of β-catenin expression and other cancer-relevant genes.

## Introduction

Heterogeneous nuclear ribonucleoproteins (hnRNPs) are an abundant family of nuclear RNA-binding proteins (RBP) that associate with nascent pre-mRNA transcripts,^1^ and have pleotropic roles in nucleic acid processing including alternative pre-RNA splicing and mRNA stability.^2^ The hnRNP-family protein structure is characterized by amine (N)-terminal RNA-recognition motifs (RRMs) as well as RGG boxes (repeats of Arg-Gly-Gly tripeptides), which aid RNA binding and can be methylated to promote nuclear export.^3^^,^^4^

Of the hnRNPs, hnRNPA2 and splice variants (hnRNPs B1/A2b/B1b, hereafter referred to collectively as hnRNPA2), encoded by the *HNRNPA2B1* gene, have generated significant interest due upregulation of expression in a number of cancers.^5^^-^^10^ Cytoplasmic localization of hnRNPA2 is associated with gastric carcinogenesis^8^ and linked with a more aggressive disease phenotype of hepatocellular carcinoma.^6^^,^^8^

Functionally, hnRNPA2 has been shown to critically mediate the cancer cell phenotype through alternative splicing of key transcripts involved in oncogenesis,^7^ tumor metabolism,^11^ invasive cell migration,^12^ and Epithelial to Mesenchymal Transition (EMT).^13^ The cytoplasmic roles of hnRNPA2 protein in oncogenesis are not characterized, but will undoubtedly be elucidated through next generation sequencing-based analyses of the RBP-regulated transcriptome.^14^

In prostate cancer (PCa), there are increasing reports of overexpression of RBPs,^15^ which are involved in a combinatorial control of alternative splicing.^16^ We previously characterized nuclear protein complexes in PCa cells associated with the RBP Sam68 (*KHDRBS1*),^17^ which is overexpressed in clinical PCa,^18^^,^^19^ and identified associations with hnRNPA2 (as well as hnRNPs A1 and L). Here, we explore the previously unreported function, expression, and localization of hnRNPA2 in PCa.

## Results

### hnRNPA2 mediates tumorigenesis and proliferation of PCa cells

To determine the role of hnRNPA2 in the tumorigenicity of PCa cells, we used RNAi to examine the colony forming efficiency of PCa cells depleted of hnRNPA2 protein. In common PCa cells lines, including the highly metastatic PC3-M cell line, hnRNPA2 protein is stably expressed (Fig. S1A), and migrates as a doublet, representing the two common isoforms. The predominant isoform in PCa cells is hnRNPA2 (bottom band) with the minor hnRNPB1 isoform (top band) expressed at very low levels. Following transfection with two different siRNA duplexes (si1 and/or si2), there was at least an ~77% reduction in hnRNPA2 protein levels compared with the non-silencing (NSi) control duplex (Fig. 1A).

**Figure:**
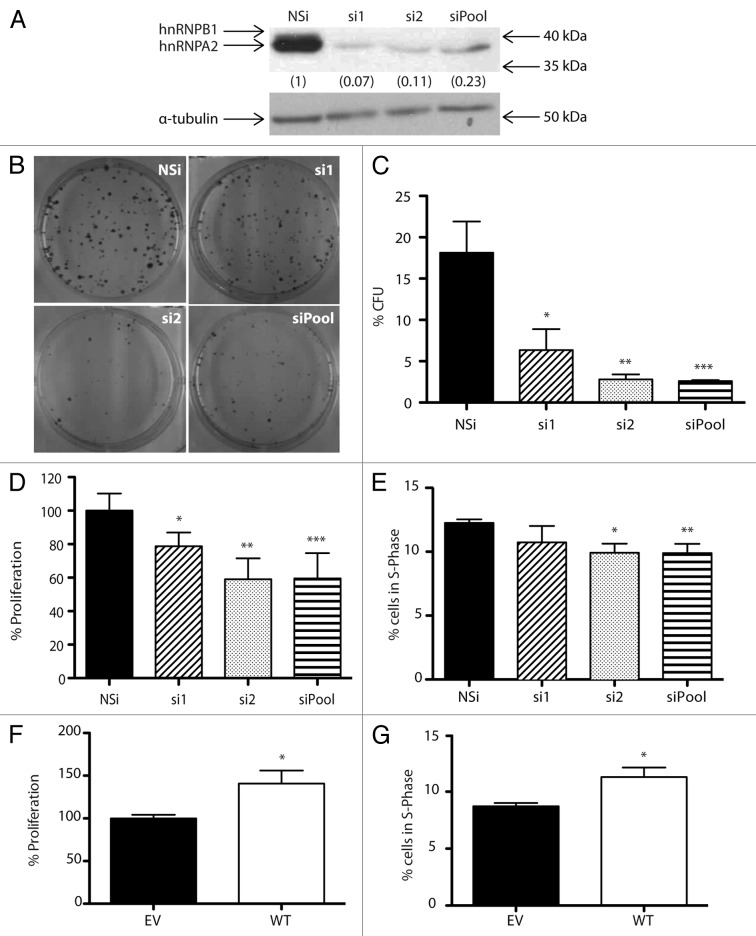
**Figure 1.** hnRNPA2 mediates tumorigenesis and proliferation of PCa cells. (**A**) PC3-M cells were transfected with two siRNA duplex sequences to hnRNPA2 (si1 or si2), or a combination (siPool), or non-silencing (NSi) control to final concentration of 25 nM. After 72 h, total cell lysates were harvested and subjected to western analysis with antibodies to hnRNPA2 and α-tubulin. Western analysis images shown are representative of three independent experiments, from which densitometric band quantitation was performed to calculate the mean relative normalized fold change in protein expression (shown in brackets). (**B and C**) Colony forming ability of PC3-M cells depleted of hnRNPA2 protein was assessed by direct cell counting (**B**), and data from at least three independent experiments were used to calculate means ± SD % colony forming efficiency (CFE) (*P = 0.03; **P = 0.008; ***P = 0.007). (**D**) Proliferation of PC3-M cells depleted of hnRNPA2 protein was measured using WST-1 proliferation reagent, and normalized to NSi control. Data from at least three independent experiments (each with at least five technical replicates) were used to calculate the means ± SE (*P = 0.048; **P = 0.01; ***P = 0.02). (**E**) Cell cycle distributions of PC3-M cells depleted of hnRNPA2 protein were assessed using flow cytometry. Percentages of cells in the S-phase of cell cycle were estimated from their DNA content as read by propidium iodine. Data from at least three independent experiments were used to calculate the means ± SD (*P = 0.03; **P = 0.03). (**F**) Proliferation of PC3 cells transfected with 0.2 µg of plasmid DNA vector encoding HA-tagged wild-type (WT) hnRNPA2 was measured using WST-1 proliferation reagent, and normalized to empty vector (EV) control. Data from at least three independent experiments with at least five technical replicates were used to calculate the means ± SE (*P = 0.002). (**G**) Cell cycle distributions of PC3 cells transfected with 2 µg of plasmid DNA vector encoding HA-tagged WT hnRNPA2 were assessed using flow cytometry, and compared with EV control. Percentages of cells in the S-phase of cell cycle were estimated from their DNA content as read by propidium iodine. Data from at least three independent experiments were used to calculate the means ± SD (*P = 0.02). (All *P *values shown are for comparisons with control conditions).

RNAi-mediated depletion of hnRNPA2 protein in PC3-M cells resulted in an ~80% reduction in the colony forming ability of the cells as compared with the non-silenced control (*P* < 0.05) (Fig. 1B and C). In the light of published data for glioblastoma cells,^7^ we hypothesized that this observed reduction in tumorigenesis was as a result of a reduction in cell proliferation. To test this hypothesis, we employed WST-1 assays to examine for changes in the proliferation of PC3-M cells depleted of hnRNPA2 with the two different siRNA duplexes (si1 and/or si2). hnRNPA2-depleted PC3-M cells exhibited an ~40% reduction in cell proliferation as compared with the non-silenced control (*P* < 0.05) (Fig. 1D).

To determine whether the above reduction in cell proliferation reflected a delay in cell cycle progression, hnRNPA2-depleted PC3-M cells were subjected to cell cycle analysis. We observed a statistically significant reduction in the proportion of PC3-M cells in S-phase (*P* < 0.05) when depleted of hnRNPA2 as compared with the non-silenced control (Fig. 1E), but we did not observe any other statistically significant changes in the proportion of cells within other stages of the cell cycle (Fig. S1B).

To test whether overexpression of hnRNPA2 protein yielded reciprocal changes in cell proliferation and cell cycle profiles, HA-tagged hnRNPA2 was ectopically expressed in PC3 cells (Fig. 1F). Following transfection with HA-tagged hnRNPA2, we observed an average of a 2.5-fold increase in hnRNPA2 protein levels compared with the empty vector control (Fig. S1C). In PC3 cells overexpressing hnRNPA2, cell proliferation was increased by 55% (*P* = 0.002) over the empty vector control (Fig. 1F). This increase in proliferation was accompanied by a statistically significant increase in the proportion of cells in S-phase (*P* = 0.02) (Fig. 1G), but no other statistically significant changes in the proportion of cells in other stages of the cell cycle were observed (Fig. S1D).

We observed similar changes to cell proliferation in the LNCaP cell line as observed in the PC3 cells for both overexpression and RNAi-mediated knockdown of hnRNPA2 (Fig. S1E and F). However, there were no associated effects on cell cycle profiles (data not shown). Taken together, these data demonstrate that hnRNPA2 protein mediates tumorigenesis and proliferation of PCa cells, possibly via activity on S-phase of the cell cycle.

### Expression of hnRNPA2 protein is upregulated in high-grade PCa

In the light of reports of upregulation of hnRNPA2 protein expression in other cancers, we examined the expression and localization of hnRNPA2 protein by immunohistochemistry, using a tissue microarray (TMA) of benign and malignant human prostate biopsies. All TMA cores demonstrated hnRNPA2 nuclear immunoreactivity within basal and luminal epithelial cells (Fig. 2A). Normality testing of Histoscores did not reveal normally distributed groups (*P* > 0.05), and therefore, further analysis was performed by non-parametric testing. There was a trend toward overexpression of nuclear hnRNPA2 protein in PCa as compared with BPH, which did not reach statistical significance (*P* = 0.067) (Table 1). Expression of nuclear hnRNPA2 expression was associated with increased pathological grade (*P* = 0.03) (Table 1 and Fig. 2). Furthermore, in a subgroup analysis of high-grade (Gleason 4 and 5) PCa, there was statistically significant upregulation of nuclear hnRNPA2 compared with low-grade (Gleason 3) PCa (*P* = 0.011), and BPH controls (*P* = 0.003).

**Figure:**
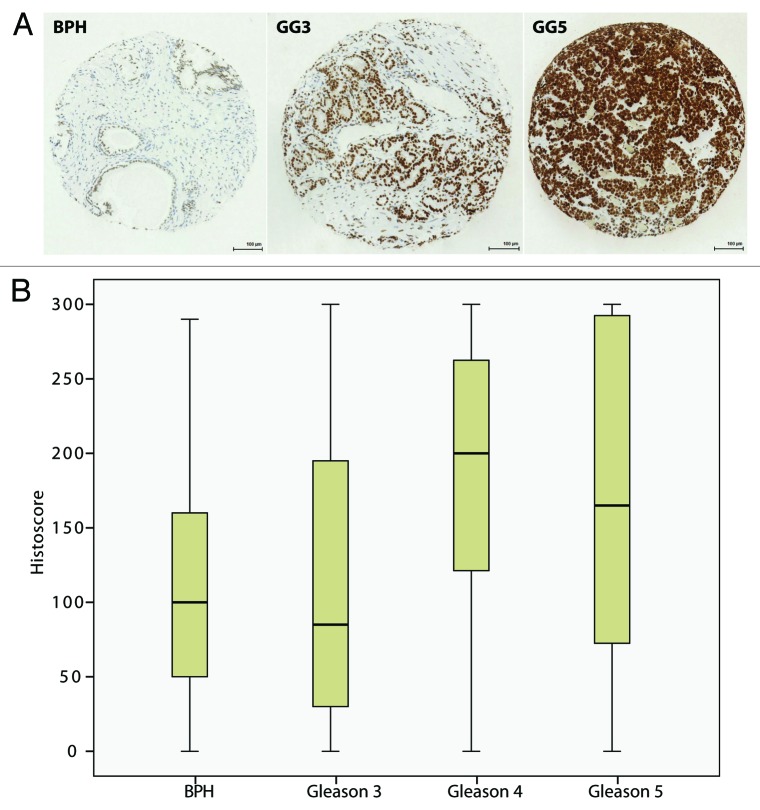
**Figure 2.** Expression of nuclear hnRNPA2 protein is upregulated in high-grade PCa. (**A**) Nuclear hnRNPA2 protein expression in clinical prostate samples. Representative images from hnRNPA2-immunostained sections for BPH (left panel), Gleason grades 3 (middle panel) and 5 (right panel). (Bar = 100 µm). (**B**) Histoscore populations are graphically represented using a box and whisker plot, as these data are not normally distributed. Statistical analysis revealed significant overexpression of nuclear hnRNPA2 protein in Gleason 4 and 5 PCa as compared with BPH controls (*P* = 0.03).

**Table:** **Table 1.** Analysis of histopathological data for nuclear hnRNPA2 protein expression

	Median histoscore ^(IQR)^	*P* value
BPH (n = 29)	100 ^(128)^	*P* = 0.069*
PCa (n = 84)	135 ^(50)^	
Gleason grade 3 (n = 41)	85 ^(166)^	*P* = 0.03**
Gleason grade 4 (n = 19)	200 ^(173)^	
Gleason grade 5 (n = 24)	165 ^(226)^	

* Mann Whitney U-Test; **Jonkheere-Terpstra; (IQR = interquartile range).

### Cytoplasmic hnRNPA2 is present in PCa cells and also drives cell proliferation

Consistent with published data for other solid organ tumors,^6^^,^^8^ immunoreactivity for hnRNPA2 protein was also observed in the cytoplasm of some PCa cores (Fig. 3A). To test whether cytoplasmic hnRNPA2 protein is also functionally relevant in PCa, we employed an ectopic expression vector encoding an HA-tagged hnRNPA2 mutant containing a deletion (R191-G253) of the RGG domain (hnRNPA2-ΔRGG), which is exclusively expressed in the cytoplasm.^4^ Expression of HA-tagged hnRNPA2-ΔRGG was confirmed by western analysis using an anti-HA-specific antibody, which detected a band migrating at a slightly lower molecular weight than wild-type HA-tagged hnRNPA2 (Fig. 3B).

**Figure:**
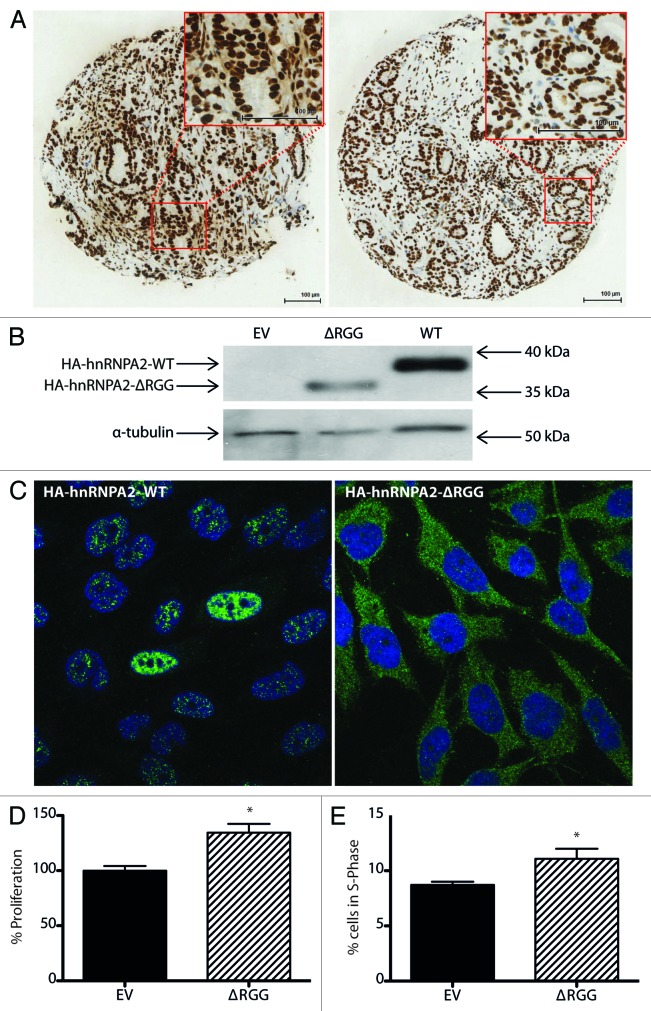
**Figure 3.** Cytoplasmic hnRNPA2 protein is expressed in clinical PCa and mediates proliferation of PCa cells. (**A**) Representative images from hnRNPA2-immunostained PCa sections demonstrating cytoplasmic and nuclear protein expression (left panel) and nuclear protein expression alone (right panel). (Bar = 100 µm). (**B**) Representative western analysis images of PC3 cells transfected with 2 µg of plasmid DNA vectors encoding HA-tagged wild-type (WT) hnRNPA2 or hnRNPA2-ΔRGG or empty vector (EV) control using antibodies to HA tag and α-tubulin. (**C**) Representative indirect immunofluorescence images of PC3 cells transfected with 2 µg of plasmid DNA vectors encoding HA-tagged WT hnRNPA2 (left) or hnRNPA2-ΔRGG (right) and captured by confocal laser scanning microscopy using indirect immunofluorescence and antibody to HA tag (green) and a DAPI nuclear counterstain (blue). (**D**) Proliferation of PC3 cells transfected with 0.2 µg of plasmid DNA vector encoding HA-tagged hnRNPA2-ΔRGG was measured using WST-1 proliferation reagent, and normalized to empty vector control. Data from at least three independent experiments with at least five technical replicates were used to calculate the means ± SE (*P = 0.02). (**E**) Cell cycle distributions of PC3 cells transfected with 2 µg of plasmid DNA vector encoding HA-tagged hnRNPA2-ΔRGG were assessed using flow cytometry. Percentages of cells in the S-phase of cell cycle were estimated from their DNA content as read by propidium iodine. Data from at least three independent experiments were used to calculate the means ± SD (*P = 0.04). (All p-values shown are for comparisons with control conditions).

To verify the cytoplasmic localization of ectopically expressed hnRNPA2, PC3 cells were transiently transfected with expression vectors for HA-tagged wild-type hnRNPA2 and hnRNPA2-ΔRGG and localization of proteins was compared by indirect immunofluorescence using the anti-HA antibody (Fig. 3C). Ectopically expressed HA-wild-type hnRNPA2 protein was predominantly present in the nuclei of PC3 cells, although was also detected at much lower levels in the cytoplasm (Fig. 3C). In contrast and consistent with previously published data,^4^ HA-hnRNPA2-ΔRGG was predominantly expressed in the cytoplasm with minimal nuclear localization.

To determine whether cytoplasmic hnRNPA2 is also functionally relevant in PCa, PC3 cells transfected transiently with HA-tagged hnRNPA2-ΔRGG were subjected to WST-1 assays and flow cytometry (Fig. 3D). In PC3 cells overexpressing hnRNPA2-ΔRGG, cell proliferation was increased by 35% (*P* = 0.02) over the empty vector control (Fig. 3D). This increase in proliferation was accompanied by a statistically significant increase in the proportion of cells in S-phase (*P* = 0.04) (Fig. 3E). Taken together, these data clearly demonstrate the presence of and function for cytoplasmic hnRNPA2 protein in PCa cells.

### hnRNPA2 acts on the 3′-UTR of CTNNB1 mRNA resulting in an increase in β-catenin protein expression

The nuclear functions of hnRNPA2 in mediating the cancer cell phenotype via alternative pre-mRNA splicing are well described,^7^^,^^11^^-^^13^ however its cytoplasmic roles are uncharacterized. In the cytoplasm, hnRNPA2 has been shown to stabilize expression of mRNAs encoded by *SLC2A1* (GLUT1) via 3′-UTR binding.^20^ Hence, we reasoned that when localized to the cytoplasm in PCa, hnRNPA2 may stabilize and/or affect translation of PCa-relevant mRNAs, which mediate its functional effects on cell proliferation.

To test this hypothesis, we searched for the association of hnRNPA2 protein with 3′-UTRs of all UCSC genes using a previously published data set of hnRNPA2 transcriptome-wide binding sites in 293T cells.^14^ To identify potential biological pathways altered by the binding of hnRNPA2 to 3′-UTR mRNAs, an enrichment analysis was performed on the gene list using the KEGG database^21^ (Table S1). We observed statistically significant enrichment for genes within “Pathways in Cancer” (n = 121/326; enrichment = 2.81-fold; FDR < 0.05), which includes a number of genes within PCa-relevant cell signaling pathways (Table S1). In particular, we identified a number of hnRNPA2 binding sites within the 3′-UTR regions of mRNAs derived from *CTNNB1* (which encodes β-catenin protein) (Fig. 4A).

**Figure:**
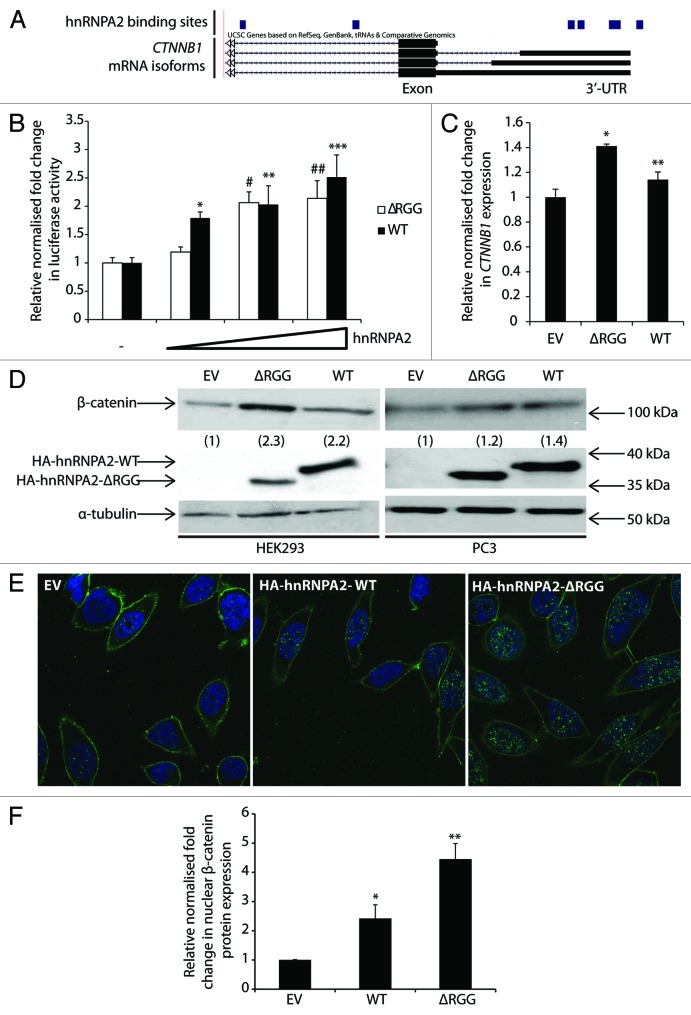
**Figure 4.** hnRNPA2 acts on the 3′-UTR of CTNNB1 mRNA resulting in an increased β-catenin protein expression. (**A**) UCSC Genome Browser showing transcript details for *CTNNB1*. Custom track showing locations of hnRNPA2 binding sites in 293T cells.^14^ (**B**) HEK293 cells were transfected with either pMiR-report-β-catenin-3′-UTR or pMiR-report-empty vector and pRL-null reporters, and HA-tagged wild-type (WT) hnRNPA2 or hnRNPA2-ΔRGG (50 ng, 100 ng, or 200 ng) or empty vector (EV). After 48 h cells were harvested for luciferase activity analysis, and data normalized to activity of pRL-null (DNA transfection control) vector as well as pMiR-report-empty (luciferase expression control) vector. Data from at least three independent experiments with at least three technical replicates were used to calculate the means ± SE (*P = 0.001, **P = 0.02, ***P = 0.009, ^#^*P* = 0.002, ^##^*P* = 0.01). (**C**) HEK293 cells transfected with 2 µg of plasmid DNA vectors encoding HA-tagged WT hnRNPA2 or hnRNPA2-ΔRGG. qRT-PCR was performed on cDNAs from two independent experiments, and levels of *CTNNB1* transcript expression were normalized to a mean of *CASC3* and *ACTB* levels to obtain mean relative normalized fold change ± SE (*P = 0.006, **P = 0.04). (**D**) HEK293 (left panel) PC3 (right panel) cells transfected with 2 µg of plasmid DNA vectors encoding HA-tagged WT hnRNPA2 or hnRNPA2-ΔRGG using antibodies to HA tag and β-catenin. Western analysis images shown are representative of three independent experiments, from which densitometric band quantitation was performed to calculate the mean relative normalized fold change in total β-catenin protein expression (shown in brackets). (**E**) PC3 cells were transfected with 2 µg of plasmid DNA vectors encoding HA-tagged WT hnRNPA2 or hnRNPA2-ΔRGG and images captured by confocal laser scanning microscopy using indirect immunofluorescence and antibody to β-catenin (green) and a DAPI nuclear counterstain (blue). Indirect immunofluorescence images shown are representative of two fields of view, from which densitometric fluorescence quantitation was performed to calculate means relative normalized fold-change in β-catenin nuclear protein expression ± SD (All *P* values shown are for comparisons with control conditions).

In the light of growing evidence implicating the activity of the Wnt/β-catenin signaling pathway in PCa,^22^ we hypothesized that the association of hnRNPA2 protein to the *CTNNB1* 3′-UTR mRNA may have an effect on mRNA stability and/or translation. To test this hypothesis, we first examined the effects of ectopic hnRNPA2 expression on 3′-UTR-mediated mRNA stability and/or translation using a luciferase reporter assay. HEK293 cells were transiently transfected with expression vectors for wild-type hnRNPA2 and hnRNPA2-ΔRGG together with a luciferase reporter containing the 3′-UTR of *CTNNB1* mRNA^23^ (Fig. 4B). Overexpression of both the wild-type and the cytoplasmic hnRNPA2-ΔRGG mutant resulted in a dose-dependent increase in luciferase reporter activity with a maximal ~2-fold enhancement in activity achieved above the stimulatory level in the absence of ectopic hnRNPA2 (*P* < 0.05).

To determine whether overexpression of hnRNPA2 protein also affected endogenous *CTNNB1* mRNA transcript expression, HEK293 cells were transiently transfected with expression vectors for wild-type hnRNPA2 and hnRNPA2-ΔRGG (Fig. 4C). Overexpression of both wild-type hnRNPA2 and the hnRNPA2-ΔRGG mutant resulted in a statistically significant increase in expression of *CTNNB1* over the empty vector control (*P* < 0.05) (Fig. 4C). To test whether an increase in mRNA expression correlated with an increase in β-catenin protein expression, whole cell lysates were subjected to western analysis using anti-HA and anti-β-catenin antibodies to confirm overexpression of HA-tagged proteins and effects on β-catenin protein expression (Fig. 4D). Overexpression of both wild-type hnRNPA2 and the hnRNP-ΔRGG mutant resulted in a dramatic 2.3-fold and 2.2-fold increase, respectively, in β-catenin protein expression over empty vector controls (Fig. 4D, left panel). These findings were confirmed in PC3 cells (Fig. 4D, right panel), although baseline β-catenin protein expression was lower than in HEK293 cells, and the fold changes observed were ~1.4-fold and ~1.2-fold for wild-type hnRNPA2 and hnRNP-ΔRGG, respectively.

Finally, we used indirect immunofluorescence to determine the localization of the increased β-catenin protein in PC3 cells following overexpression of hnRNPA2 and hnRNPA2-ΔRGG (Fig. 4E). In the absence of ectopic hnRNPA2 expression, β-catenin protein was predominantly localized to the cell membrane, with some nuclear expression. Ectopic expression of both wild-type hnRNPA2 and the hnRNPA2-ΔRGG resulted in a statistically significant increase in nuclear expression of β-catenin protein (*P* < 0.05) (Fig. 4F). Taken together, our data demonstrate that hnRNPA2 acts on the 3′-UTR of *CTNNB1* mRNA resulting in an increase in *CTNNB1* mRNA stability, β-catenin protein expression, and nuclear localization.

## Discussion

Nuclear hnRNPA2 protein is overexpressed in cancer and has been shown to mediate the cancer cell phenotype through alternative pre-mRNA splicing.^7^^,^^11^^-^^13^ Cytoplasmic localization of hnRNPA2 is reported in aggressive cancers,^6^^,^^8^ although its specific functional role in cancer is not yet characterized. Here, we demonstrate overexpression of hnRNPA2 protein in high-grade PCa as enhanced hnRNPA2 immunoreactivity, which is in keeping with data for other solid organ tumors.^6^^,^^8^ As the antibody to hnRNPA2 recognizes both the A2 and B1 isoforms of this protein, we were unable to determine the relative contributions of these two isoforms to overall protein expression by immunohistochemistry. However, by western analysis of human PCa cell lines, the A2 isoform appears predominant (Fig. S1A). Future evaluation of these isoforms in clinical PCa samples would be of interest.

Consistent with published data,^7^^,^^13^ we also demonstrate that PCa cells depleted of hnRNPA2 have diminished proliferative and tumorigenic potential. Additionally we report, for the first time, a reciprocal increase in cell proliferation through overexpression of a solely cytoplasmic hnRNP-A2-ΔRGG mutant in PCa cells. In keeping with the cytoplasmic functions of hnRNPs,^2^ we identified a novel role for hnRNPA2 protein in *CTNNB1* mRNA 3′-UTR-mediated mRNA stability, and expression and nuclear localization of β-catenin protein, where it is thought to exert its transcriptional effects to drive tumorigenesis.^24^ Although the mechanisms of β-catenin protein degradation are well-described, comparatively little is known about the regulation of its production.^24^ Consistent with our findings, others have shown that RBPs can modulate *CTNNB1* mRNA metabolism and β-catenin protein expression.^25^^-^^29^ Although a previously published study did not identify a decrease in β-catenin protein expression in hnRNPA2-depleted cells,^7^ this may be due to functional redundancy as exemplified by complex mRNA transcript cross- and auto-regulation by hnRNP paralogs.^14^^,^^30^ In cancer, where RBPs are typically overexpressed, the biological significance of this phenomenon is unclear.

There is increasing evidence for a greater importance of mRNA and protein metabolism in the quantitative control of gene expression.^31^ Conserved mRNA 3′-UTR c*is*-regulatory regions can influence polyadenylation, localization, stability, and translation under the control of *trans*-acting factors such as miRNAs and RBPs,^32^ and reprogramming of mRNA 3′-UTRs can affect cell phenotypes.^33^ Hence, in PCa, overexpression and cytoplasmic localization of hnRNPA2 protein may increase *CTNNB1* mRNA stability and/or protein expression via direct 3′-UTR binding, thereby contributing to cell proliferation.

Our KEGG analysis of a published transcriptome-wide footprint of hnRNPA2^21^ revealed hnRNPA2 binding sites within the 3′-UTRs of several cancer-relevant genes. Although the specific functional consequences of these RBP-mRNA associations remain unclear, it is likely that hnRNPA2 protein is involved in 3′-UTR-mediated mRNA stability and translation of a number of other cancer-relevant genes in addition to *CTNNB1*. Further transcriptome-wide mapping studies of hnRNPA2 binding sites in the context of specific PCa phenotypes and downstream functional analyses are required to fully elucidate the role of this RBP in prostate tumorigenesis and PCa disease progression.

## Materials and Methods

### Antibodies, plasmids, and oligonucleotides

The following antibodies were used: anti-hnRNPA2B1 (Abcam; DP3B3), anti-α-tubulin (TU-02, Santa Cruz Biotechnology) anti-β-catenin (C19220, BD Biosciences), anti-HA (haemagglutinin)-Tag (6E2, Cell Signaling), anti-mouse IgG HRP-linked (7076, Cell Signaling), Alexa Fluor® 488 anti-Mouse IgG (A-11001, Invitrogen). The following plasmids have been described previously: pcDNA3-HA-hnRNPA2- cDNA,^34^ pCAGPM-HA-hnRNPA2,^35^ pMiR-Report -3′-UTR- β-catenin.^23^ Sequences used to generate siRNA duplexes were as previously described^12^: si1 5′-GAAUUAUUUA AUAACAUUA-3′ and si2 5′-GAAGAGUAGU UGAGCCAAA-3′, and a non-silencing control (# D-001810-01-20, Dharmacon) was also used. Sequences used to generate oligonucleotide primers for PCR were as follows: CTNNB1_F 5′-gctttcagtt gagctgacca-3′ and CTNNB1_R 5′-caagtccaag atcagcagtc tc-3′, CASC3_F 5′-ggggttccag ttaatacaag tttc-3′ and CASC3_r 5′-gccagctgta tttctcttct gag-3′, and, ACTB_F 5’-attggcaatgagcggttc-3’ and ACTB_R 5’-cgtggatgccacaggact-3’. Universal Probe Library (04683633001, Roche) short hydrolysis probes 21 (CTNNB1), 84 (CASC3), and 11 (ACTB) were used for qPCR analysis.

### Cell culture, DNA, and siRNA transfections

All cells were grown at 37 °C in 5% CO_2_. LNCaP (CRL-1740, ATCC), PC3 (CRL-1435, ATCC), and HEK293 (CRL-1573, ATCC) cells were maintained in RPMI-1640 medium (31870-025, Life Technologies) with 2 mM L-glutamine (25030-024, Life Technologies), supplemented with 10% fetal bovine serum (FBS) (A15-101, PAA Laboratories). PC3-M cells were derived as previously described^36^ and maintained as above. Transfections with plasmid DNA were performed as detailed in the figure legends using Lipofectamine LTX (15338-100, Life Technologies) according to manufacturer’s instructions. Transfections with siRNA duplexes were performed as detailed in the figure legends using RNAiMax (13778-075, Life Technologies) according to manufacturer’s instructions. Data obtained from siRNA-mediated silencing of hnRNPA2 were normalized to those from the non-silencing control (# D-001810-01-20, Dharmacon).

### Western analysis

Whole cell lysis was performed in sample loading buffer (0.125 M Tris pH 6.8, 2% SDS, 10% glycerol, 10% β-mercaptoethanol, and 0.01% bromophenol blue) prior to protein fractionation by SDS-Polyacrylamide Gel Electrophoresis (SDS-PAGE) on 12% w/v Tris gels. Electrophoresed samples were electroblotted onto an Immobilon-P membrane (IPVH00010, Millipore) prior to western analysis. Antibody concentrations were as follows: anti-hnRNPA2B1 (1:1000), anti-HA (1:1000), anti-α-tubulin (1:1000). HRP-linked secondary antibody (1:2000) was used for signal detection using Amersham ECL Western Blotting Detection Reagent (RPN2232, GE Life Sciences). Densitometric assessment of protein bands from at least three independent experiments was performed using ImageJ (http://rsb.info.nih.gov/ij/), and intensities used to calculate mean relative normalized fold-change in protein expression.

### Proliferation assays

Proliferation assays were performed using the WST-1 cell proliferation reagent (05015944001, Roche) as per manufacturer's instructions. Briefly, 4000–10 000 cells were seeded into each well of a 96-well plates and grown to ~20–30% confluence prior to transfection with either siRNA or DNA as indicated above. After 72 h, 10 μl WST-1 reagent was added to each well, mixed, and left to develop for 1 h before reading the absorbance at 450 nm (with reference wavelength at 650 nm) using SpectraMax Plus384 Absorbance Microplate Reader (Molecular Devices). All data were normalized to either a non-silencing control or an empty vector control. Results shown are the means ± SEM of at least three independent experiments with at least five technical replicates.

### Colony forming assays

Cells were seeded at a density of 500 cells/well into 6-well plates. After 14 d, colonies were fixed in methanol for 20 min, stained with hematoxylin, photographed using GeneSnap v.1.0 (Syngene), and counted. Percentage colony forming efficiency (CFE) was calculated as follows: Colonies counted/number of cells seeded × 100. Results shown are the means ± SD of at least three independent experiments.

### Flow cytometry

For cell cycle analysis, cells harvested and re-suspended in PBS (phosphate-buffered saline) containing 2% FBS, permeabilized with 1% Triton X-100, stained with 2.5 μg/μl propidium iodide (81845, Fluka), and treated with RNase (100 μg/ml), at least 10 000 cells were evaluated for each sample. Data were collected and analyzed using CellQuest Pro software (337452, BD Biosciences) on a FACScan cytometer (337452, BD Biosciences). Percentages of cells in the different stages of the cell cycle were estimated from their DNA content as read by propidium iodine. Results shown are the means ± SD of at least three independent experiments.

### Immunohistochemistry

Immunohistochemistry was performed using a tissue microarray (TMA) of 30 benign and 122 treatment-naive malignant prostate biopsies derived by transurethral resection (TUR), as previously described^37^ in accordance with ethical approval (Trent Multi-center Research Ethics Committee Ref: MREC/01/4/061). Anti-hnRNPA2 antibody was used at a concentration of 1:1200 following optimization of antibody dilution on tissue blocks. hnRNPA2 nuclear immunoreactivity was scored blindly with shielding from clinical data using a modification of the weighted histoscore method:^38^ Histoscores were calculated from the sum of (1 × % cells staining weakly) + (2 × % moderately positive) + (3 × % cells staining strongly positive), with a maximum of 300. Consistent with previously published data,^39^ our TMA analysis failure was 26% due to technical issues (loss of cores, change in pathology). The final study included 84 PCa and 29 BPH (Benign Prostatic Hyperplasia) cores. The mean of the two histoscores obtained from assessment of immunoreactivity of two technical replicates was used for statistical analysis. All images were captured using ScanScope CS scanner (Aperio) and viewed using the Slidepath Gateway viewer (Leica).

### Immunofluorescence

Cells were cultured in 6-well plates on glass coverslips, fixed with 100% methanol for 20 min at -20 °C, permeabilized in 0.1% Triton X-100, and blocked with 10% goat serum in PBS. Cells were incubated with the primary antibody and subsequently fluorescent probe-linked secondary antibody. Finally, coverslips were mounted with Vectrashield Mounting Medium (H-1200, Vector Laboratories) containing DAPI (4’, 6-diamidino-2-phenylindole) DNA counterstain. All images were captured using confocal laser scanning microscopy (LSM510, Zeiss). Typically, 50–70% cell transfection efficiency was observed for tagged constructs. Densitometric assessment of cell fluorescence from two fields of view at x63 magnification was performed using ImageJ (http://rsb.info.nih.gov/ij/), and intensities used to calculate means ± SD relative normalized fold-change in protein expression.

### Bioinformatics

Publically available sequence read archive (SRA) files containing transcriptome-wide binding sites for hnRNPA2B1^14^ were uploaded to Galaxy^40^ using the EBI-SRA tool, and mapped to the human genome assembly hg18 using Bowtie2.^41^ Intervals overlapping with 3′-UTRs derived from University of California Santa Cruz (UCSC) known genes were intersected and uploaded into the UCSC Genome Browser.^42^ Enriched KEGG (Kyoto Encyclopedia of Genes and Genomes) pathways^21^ were identified by uploading the final gene list of transcripts with hnRNPA2 binding sites within their 3′-UTRs to WebGestalt (WEB-based GEne SeT AnaLysis Toolkit).^43^ Associations and testing of each pathway for gene enrichment was performed through the WebGestalt software with a hypergeometric test using the Bonferroni correction method for multiple testing. Pathways were deemed to be enriched if the ratio of enrichment over background was at least 2-fold and FDR < 0.05.

### Luciferase reporter assays

HEK293 cells were seeded at a density of 2 × 10^4^ cells/well in 24-well plates. Cells were transfected with DNA as detailed in the figure legends. Firefly and Renilla luciferase assays were performed using the Dual Luciferase Reporter Assay system (E1910, Promega) as per manufacturer’s instructions to give relative luciferase activity. Results shown are the means ± SE of at least three independent experiments with three technical replicates.

### RNA extraction, reverse transcription (RT), and quantitative PCR (PCR)

Total RNA was extracted using the RNeasy Mini Kit (74104, QIAGEN) according to the manufacturer’s instructions. Reverse transcription of 1 µg of total RNA using the High-Capacity cDNA Reverse Transcription Kit (4368814, Applied Biosystems) according to the manufacturer’s instructions. qPCR was performed on the 7500 Fast Real-Time PCR machine (Applied Biosystems, 4351106) using triplicate cDNA templates with the TaqMan Universal PCR Master mix (4304437, Roche Diagnostics) and Universal Probe Library set (04683633001, Roche) according to the manufacturer’s instructions. Reaction conditions were as follows: 20 s at 50 °C, 10 min at 95 °C, and 40 cycles of 15 s at 95 °C and 1 min at 60 °C. Relative gene expression was determined by the 2^-ΔΔCT^ method using SDS v1.4.2 software (Applied Biosystems) using the mean of two validated endogenous control genes (*CASC3* and *ACTB*) to ensure the reliability and reproducibility of observed effects. All data was normalized to the empty vector controls. Results shown are the means ± SE of two independent experiments with three technical replicates.

### Statistical analysis

The one-sample Kolmogorov–Smirnov test was used for assessment of the normality of clinical data, and Mann–Whitney U-test and Jonkheere–Terpstra test employed to identify differences between groups. For in vitro data, the one-tailed independent sample T-Test was employed to identify differences between groups. All statistical tests were undertaken using SPSS v.19.0 (SPSS, Inc.) and Prism v.6 (Graphpad) computer software with *P* < 0.05 taken to indicate statistical significance.

## Supplementary Material

Additional material

## Notes

10.4161/rna.28800

## References

[1] Dreyfuss G, Matunis MJ, Piñol-Roma S, Burd CG (1993). hnRNP proteins and the biogenesis of mRNA. Annu Rev Biochem.

[2] Han SP, Tang YH, Smith R (2010). Functional diversity of the hnRNPs: past, present and perspectives. Biochem J.

[3] Liu Q, Dreyfuss G (1995). In vivo and in vitro arginine methylation of RNA-binding proteins. Mol Cell Biol.

[4] Nichols RC, Wang XW, Tang J, Hamilton BJ, High FA, Herschman HR, Rigby WF (2000). The RGG domain in hnRNP A2 affects subcellular localization. Exp Cell Res.

[5] Chen ZY, Cai L, Zhu J, Chen M, Chen J, Li ZH, Liu XD, Wang SG, Bie P, Jiang P (2011). Fyn requires HnRNPA2B1 and Sam68 to synergistically regulate apoptosis in pancreatic cancer. Carcinogenesis.

[6] Cui H, Wu F, Sun Y, Fan G, Wang Q (2010). Up-regulation and subcellular localization of hnRNP A2/B1 in the development of hepatocellular carcinoma. BMC Cancer.

[7] Golan-Gerstl R, Cohen M, Shilo A, Suh SS, Bakàcs A, Coppola L, Karni R (2011). Splicing factor hnRNP A2/B1 regulates tumor suppressor gene splicing and is an oncogenic driver in glioblastoma. Cancer Res.

[8] Jing GJ, Xu DH, Shi SL, Li QF, Wang SY, Wu FY, Kong HY (2011). Aberrant expression and localization of hnRNP-A2/B1 is a common event in human gastric adenocarcinoma. J Gastroenterol Hepatol.

[9] Zhou J, Allred DC, Avis I, Martínez A, Vos MD, Smith L, Treston AM, Mulshine JL (2001). Differential expression of the early lung cancer detection marker, heterogeneous nuclear ribonucleoprotein-A2/B1 (hnRNP-A2/B1) in normal breast and neoplastic breast cancer. Breast Cancer Res Treat.

[10] Zhou J, Mulshine JL, Unsworth EJ, Scott FM, Avis IM, Vos MD, Treston AM (1996). Purification and characterization of a protein that permits early detection of lung cancer. Identification of heterogeneous nuclear ribonucleoprotein-A2/B1 as the antigen for monoclonal antibody 703D4. J Biol Chem.

[11] David CJ, Chen M, Assanah M, Canoll P, Manley JL (2010). HnRNP proteins controlled by c-Myc deregulate pyruvate kinase mRNA splicing in cancer. Nature.

[12] Moran-Jones K, Grindlay J, Jones M, Smith R, Norman JC (2009). hnRNP A2 regulates alternative mRNA splicing of TP53INP2 to control invasive cell migration. Cancer Res.

[13] Tauler J, Zudaire E, Liu H, Shih J, Mulshine JL (2010). hnRNP A2/B1 modulates epithelial-mesenchymal transition in lung cancer cell lines. Cancer Res.

[14] Huelga SC, Vu AQ, Arnold JD, Liang TY, Liu PP, Yan BY, Donohue JP, Shiue L, Hoon S, Brenner S (2012). Integrative genome-wide analysis reveals cooperative regulation of alternative splicing by hnRNP proteins. Cell Rep.

[15] Rajan P, Elliott DJ, Robson CN, Leung HY (2009). Alternative splicing and biological heterogeneity in prostate cancer. Nat Rev Urol.

[16] Matlin AJ, Clark F, Smith CW (2005). Understanding alternative splicing: towards a cellular code. Nat Rev Mol Cell Biol.

[17] Rajan P, Dalgliesh C, Bourgeois CF, Heiner M, Emami K, Clark EL, Bindereif A, Stevenin J, Robson CN, Leung HY (2009). Proteomic identification of heterogeneous nuclear ribonucleoprotein L as a novel component of SLM/Sam68 Nuclear Bodies. BMC Cell Biol.

[18] Rajan P, Gaughan L, Dalgliesh C, El-Sherif A, Robson CN, Leung HY, Elliott DJ (2008). The RNA-binding and adaptor protein Sam68 modulates signal-dependent splicing and transcriptional activity of the androgen receptor. J Pathol.

[19] Busà R, Paronetto MP, Farini D, Pierantozzi E, Botti F, Angelini DF, Attisani F, Vespasiani G, Sette C (2007). The RNA-binding protein Sam68 contributes to proliferation and survival of human prostate cancer cells. Oncogene.

[20] Hamilton BJ, Nichols RC, Tsukamoto H, Boado RJ, Pardridge WM, Rigby WF (1999). hnRNP A2 and hnRNP L bind the 3’UTR of glucose transporter 1 mRNA and exist as a complex in vivo. Biochem Biophys Res Commun.

[21] Kanehisa M, Goto S, Kawashima S, Okuno Y, Hattori M (2004). The KEGG resource for deciphering the genome. Nucleic Acids Res.

[22] Kypta RM, Waxman J (2012). Wnt/beta-catenin signalling in prostate cancer. Nat Rev Urol.

[23] Hsieh IS, Chang KC, Tsai YT, Ke JY, Lu PJ, Lee KH, Yeh SD, Hong TM, Chen YL (2013). MicroRNA-320 suppresses the stem cell-like characteristics of prostate cancer cells by downregulating the Wnt/beta-catenin signaling pathway. Carcinogenesis.

[24] Clevers H, Nusse R (2012). Wnt/β-catenin signaling and disease. Cell.

[25] Bikkavilli RK, Malbon CC (2010). Dishevelled-KSRP complex regulates Wnt signaling through post-transcriptional stabilization of beta-catenin mRNA. J Cell Sci.

[26] Gherzi R, Trabucchi M, Ponassi M, Ruggiero T, Corte G, Moroni C, Chen CY, Khabar KS, Andersen JS, Briata P (2006). The RNA-binding protein KSRP promotes decay of beta-catenin mRNA and is inactivated by PI3K-AKT signaling. PLoS Biol.

[27] Ruggiero T, Trabucchi M, Ponassi M, Corte G, Chen CY, al-Haj L, Khabar KS, Briata P, Gherzi R (2007). Identification of a set of KSRP target transcripts upregulated by PI3K-AKT signaling. BMC Mol Biol.

[28] Yang G, Fu H, Zhang J, Lu X, Yu F, Jin L, Bai L, Huang B, Shen L, Feng Y (2010). RNA-binding protein quaking, a critical regulator of colon epithelial differentiation and a suppressor of colon cancer. Gastroenterology.

[29] Fu Y, Huang B, Shi Z, Han J, Wang Y, Huangfu J, Wu W (2013). SRSF1 and SRSF9 RNA binding proteins promote Wnt signalling-mediated tumorigenesis by enhancing β-catenin biosynthesis. EMBO Mol Med.

[30] Spellman R, Llorian M, Smith CW (2007). Crossregulation and functional redundancy between the splicing regulator PTB and its paralogs nPTB and ROD1. Mol Cell.

[31] Schwanhäusser B, Busse D, Li N, Dittmar G, Schuchhardt J, Wolf J, Chen W, Selbach M (2011). Global quantification of mammalian gene expression control. Nature.

[32] Matoulkova E, Michalova E, Vojtesek B, Hrstka R (2012). The role of the 3′ untranslated region in post-transcriptional regulation of protein expression in mammalian cells. RNA Biol.

[33] Li J, Lu X (2013). The emerging roles of 3′ untranslated regions in cancer. Cancer Lett.

[34] Ferron L, Davies A, Page KM, Cox DJ, Leroy J, Waithe D, Butcher AJ, Sellaturay P, Bolsover S, Pratt WS (2008). The stargazin-related protein gamma 7 interacts with the mRNA-binding protein heterogeneous nuclear ribonucleoprotein A2 and regulates the stability of specific mRNAs, including CaV2.2. J Neurosci.

[35] Katoh H, Mori Y, Kambara H, Abe T, Fukuhara T, Morita E, Moriishi K, Kamitani W, Matsuura Y (2011). Heterogeneous nuclear ribonucleoprotein A2 participates in the replication of Japanese encephalitis virus through an interaction with viral proteins and RNA. J Virol.

[36] Pettaway CA, Pathak S, Greene G, Ramirez E, Wilson MR, Killion JJ, Fidler IJ (1996). Selection of highly metastatic variants of different human prostatic carcinomas using orthotopic implantation in nude mice. Clin Cancer Res.

[37] Ahmad I, Patel R, Singh LB, Nixon C, Seywright M, Barnetson RJ, Brunton VG, Muller WJ, Edwards J, Sansom OJ (2011). HER2 overcomes PTEN (loss)-induced senescence to cause aggressive prostate cancer. Proc Natl Acad Sci U S A.

[38] Kirkegaard T, Edwards J, Tovey S, McGlynn LM, Krishna SN, Mukherjee R, Tam L, Munro AF, Dunne B, Bartlett JM (2006). Observer variation in immunohistochemical analysis of protein expression, time for a change?. Histopathology.

[39] Torhorst J, Bucher C, Kononen J, Haas P, Zuber M, Köchli OR, Mross F, Dieterich H, Moch H, Mihatsch M (2001). Tissue microarrays for rapid linking of molecular changes to clinical endpoints. Am J Pathol.

[40] Blankenberg D, Von Kuster G, Coraor N, Ananda G, Lazarus R, Mangan M, Nekrutenko A, Taylor J. Galaxy: a web-based genome analysis tool for experimentalists. Current protocols in molecular biology / edited by Frederick M Ausubel [et al] 2010; Chapter 19:Unit 19 0 1-21. 10.1002/0471142727.mb1910s89 PMC4264107 20069535

[41] Langmead B, Trapnell C, Pop M, Salzberg SL (2009). Ultrafast and memory-efficient alignment of short DNA sequences to the human genome. Genome Biol.

[42] Kent WJ, Sugnet CW, Furey TS, Roskin KM, Pringle TH, Zahler AM, Haussler D (2002). The human genome browser at UCSC. Genome Res.

[43] Wang J, Duncan D, Shi Z, Zhang B (2013). WEB-based GEne SeT AnaLysis Toolkit (WebGestalt): update 2013. Nucleic Acids Res.

