# Supplementary material for: The RNA-binding protein hnRNPA2 regulates β-catenin protein expression and is overexpressed in prostate cancer
Source: RNA Biol. 2014 Apr 24;11(6):755–65. doi: 10.4161/rna.28800 (PMC4156506; doi:10.4161/rna.28800)
Supplement: Additional material [file rna-11-755-s01.pdf]

Landes Bioscience
www.landesbioscience.com

Supplemental Material to:

Jacqueline Stockley, M Eugenia M Martin, Colin Nixon, Imran Ahmad, Hing Y Leung, and Prabhakar Rajan

The RNA-binding protein hnRNPA2 regulates β-catenin protein expression and is overexpressed in prostate cancer

2014; 11(6)
http://dx.doi.org/10.4161/rna.28800

www.landesbioscience.com/journals/rnabiology/article/28800/

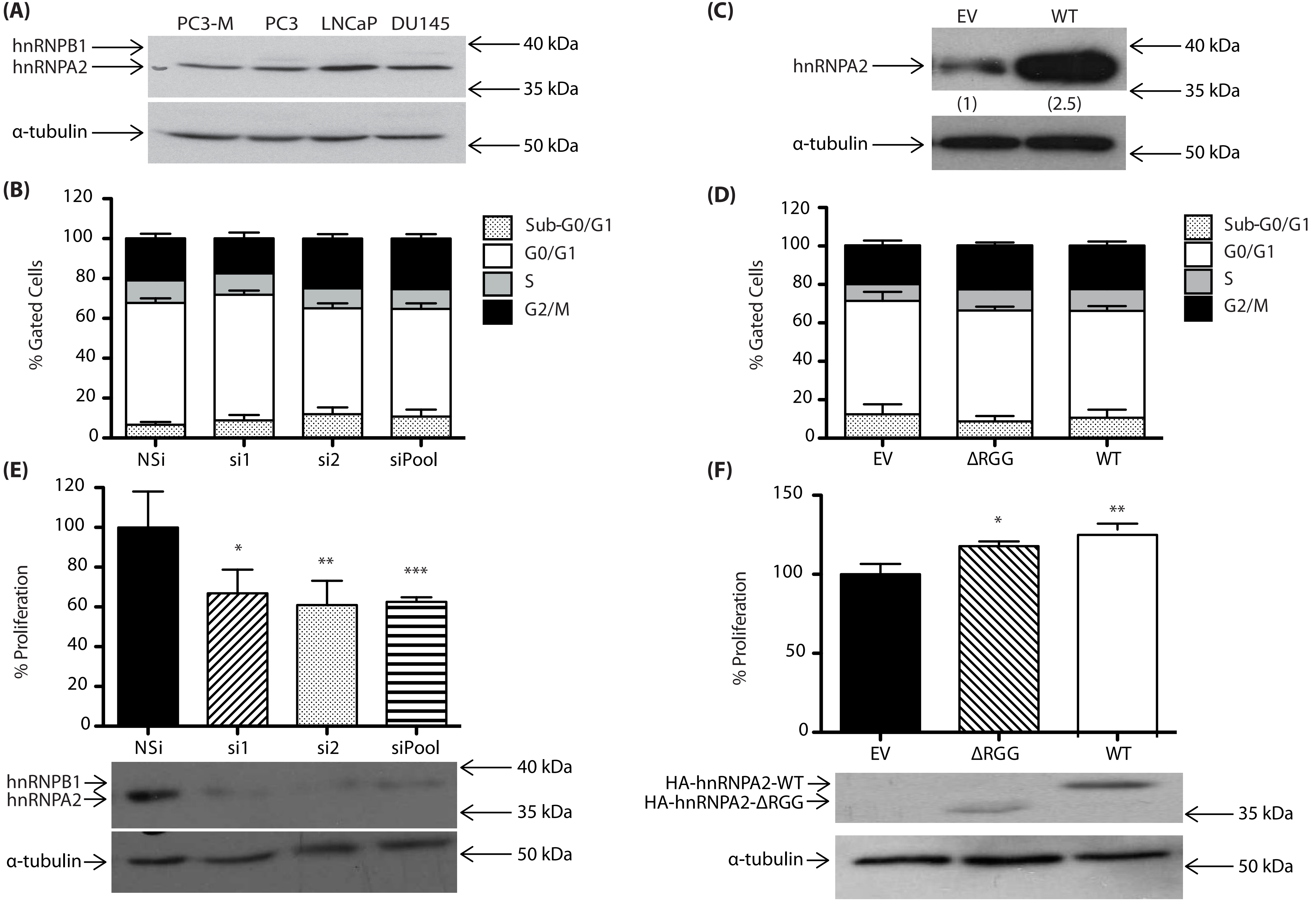

Supplementary Figure S1

Term	Description	Genes with 3'UTR hnRNPA2B1 binding sites	Genes in pathway	Fold enrichment	FDR
hsa01100	Metabolic pathways	330	1130	2.21	2.96E-44
hsa04141	Protein processing in endoplasmic reticulum	89	165	4.09	1.58E-33
hsa04120	Ubiquitin mediated proteolysis	72	135	4.04	8.87E-27
hsa05200	Pathways in cancer	121	326	2.81	4.22E-26
hsa03013	RNA transport	74	151	3.71	1.07E-24
hsa03040	Spliceosome	64	127	3.82	2.85E-22
hsa04722	Neurotrophin signaling pathway	60	127	3.58	4.02E-19
hsa03010	Ribosome	49	92	4.03	1.71E-18
hsa04810	Regulation of actin cytoskeleton	80	213	2.85	6.85E-18
hsa04110	Cell cycle	57	124	3.48	1.29E-17

Supplemental information

Supplementary Figure S1. Nuclear and cytoplasmic hnRNPA2 mediate proliferation of PCa cells. (A) Western analysis of PCa cell lines grown in steady-state conditions using antibodies to hnRNPA2 and α-tubulin. (B) Cell cycle distributions of PC3 cells silenced for hnRNPA2 protein were assessed using flow cytometry. Percentages of cells in phases of the cell cycle were estimated from their DNA content as read by propidium iodine. (C) Representative western analysis images of PC3 cells transfected with 2 µg of plasmid DNA vectors encoding HA-tagged wild-type (WT) hnRNPA2 or empty vector (EV) using antibodies to hnRNPA2 and α-tubulin. Densitometric band quantitation was performed to calculate the mean relative normalised fold change in protein expression (shown in brackets). (D) Cell cycle distributions of PC3 cells transfected with 2 µg of plasmid DNA vectors encoding HA-tagged WT hnRNPA2 or hnRNPA2-ΔRGG were assessed using flow cytometry. Percentages of cells in phases of the cell cycle were estimated from their DNA content as read by propidium iodine. (E) Proliferation of LNCaP cells silenced for hnRNPA2 protein was measured using WST-1 proliferation reagent, and normalised to NSi control (*p=0.02, **p=0.01, ***p=0.01). Total cell lysates were harvested and subjected to western analysis with antibodies to hnRNPA2 and α-tubulin. (F) Proliferation of LNCaP cells transfected with 0.2 µg of plasmid DNA vectors encoding HA-tagged WT hnRNPA2 or hnRNPA2-ΔRGG was measured using WST-1 proliferation reagent, and normalised to EV control (*p=0.04, **p=0.009). Total cell lysates were harvested and subjected to western analysis with antibodies to HA and α-tubulin. (All p-values shown are for comparisons with control conditions).

Supplementary Table S1. Enriched KEGG pathways. Pathways enriched at least 2-fold with an FDR<0.05.
